# Hypercapnia from Physiology to Practice

**DOI:** 10.1155/2022/2635616

**Published:** 2022-09-23

**Authors:** Amilkar Almanza-Hurtado, Camilo Polanco Guerra, María Cristina Martínez-Ávila, Diana Borré-Naranjo, Tomás Rodríguez-Yanez, Carmelo Dueñas-Castell

**Affiliations:** ^1^Intensive Care Unit, Universidad de Cartagena, Cartagena, Colombia; ^2^Medicine Program, Universidad del Sinu, Cartagena, Colombia; ^3^Epidemiology and Public Health, BIOTOXAM Group, Cartagena, Colombia

## Abstract

Acute hypercapnic ventilatory failure is becoming more frequent in critically ill patients. Hypercapnia is the elevation in the partial pressure of carbon dioxide (PaCO_2_) above 45 mmHg in the bloodstream. The pathophysiological mechanisms of hypercapnia include the decrease in minute volume, an increase in dead space, or an increase in carbon dioxide (CO_2_) production per sec. They generate a compromise at the cardiovascular, cerebral, metabolic, and respiratory levels with a high burden of morbidity and mortality. It is essential to know the triggers to provide therapy directed at the primary cause and avoid possible complications.

## 1. Introduction

Carbon dioxide (CO_2_) is a gas and metabolic product that influences several cellular processes, including respiration, the affinity of hemoglobin for oxygen, and regulation of blood pH and acid-based balance [1]. Healthy people can regulate CO_2_ levels by negative feedback mechanisms modulated by central and peripheral chemoreceptors [2, 3]. Hypercapnia is the elevation in the partial pressure of carbon dioxide (PaCO_2_) above 45 mmHg [4].

Carbon dioxide levels alter in response to alveolar ventilation (VA) changes caused by respiratory depression, airway obstruction, and increased dead space (VD), reflecting excessive production of CO_2_, inadequate elimination of the same [5–7], or a neuromuscular disorder. Hypercatabolic states, sepsis, thyrotoxicosis, overfeeding, and malignant hyperthermia can elevate CO_2_ levels; each degree Celsius increase in body temperature increases CO_2_ production by 14% [8].

Hypercapnia in critically ill patients is related to readmission to the intensive care unit and increased in-hospital and 30-day mortality [9]. Many patients affected by various respiratory diseases may develop hypercapnia, for instance, obesity, obesity-hypoventilation syndrome, COPD, sleep apnea, and neuromuscular disorders, among others, in the acute or chronic phase, which contributes during their hospitalization to the appearance of severe hypercapnic respiratory failure.

The objective of this review is to summarize the pathophysiological aspects of hypercapnia in critically ill patients and, based on them, offer a diagnostic, therapeutic, and preventive approach to impact fewer complications and better clinical outcomes.

## 2. Physiology of CO_2_: Production and Diffusion of CO_2_

In 1776, Lavoisier said that “of all the phenomena of the animal economy, there is not one more surprising or worthy of the attention of philosophers and physiologists than those that accompany respiration” [10]. Even though there was a brief description of carbon dioxide by van Helmont (1580–1644) [11], it took more than a century for what should now be recognized as the discovery of CO_2_, published in 1754 by Josephus Black in his inaugural medical dissertation on “humore acido a cibis ortho, et magnesia alba” [12].

**Figure 1:**
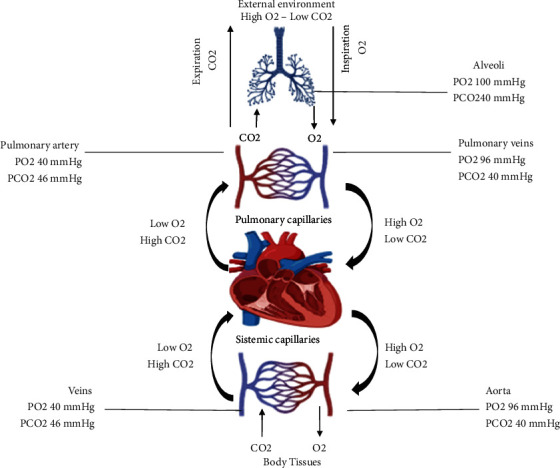
Gas exchange. Figure adapted from [16].

Carbon dioxide is a gas and metabolic product that influences several cellular processes, including respiration, the affinity of hemoglobin for oxygen, and the regulation of blood pH and acid-based balance [13]. Additionally, it has potent vascular and immunological effects [14, 15]. CO_2_ is a small molecule that crosses biological cell membranes by passive diffusion, depending on the transmembrane concentration gradient of CO_2_ and the gas's lipid/water partitioning behavior. Once inside the cell, CO_2_ equilibrates with its hydrated form, H_2_CO_3_, rapidly dissociating into H+ and HCO_3_, catalyzed by carbonic anhydrase [13]. It is possible that under extreme circumstances, such as severe ischemia, thyroid crisis, hypoxia, sepsis, highly high metabolic rates (such as during maximal exercise), or severe acid-base disturbances, the final product might be strongly favored over other products [8]. Healthy people can regulate CO_2_ levels by negative feedback mechanisms modulated by central and peripheral chemoreceptors [2, 3] (Figure 1).

One of the purposes of respiration is to remove CO_2_ from the body through gas diffusion. Under normal conditions, the diffusion capacity is 25 ml/min/mmHg and can increase with effort [17]. Fick's laws of diffusion state that the amount of gas transferred through a sheet of tissue is directly proportional to the exchange surface (A), the diffusion constant (D), and the partial pressure difference (P1−P2) and inversely proportional to its thickness (T) [17] (Table 1).

**Figure 2:**
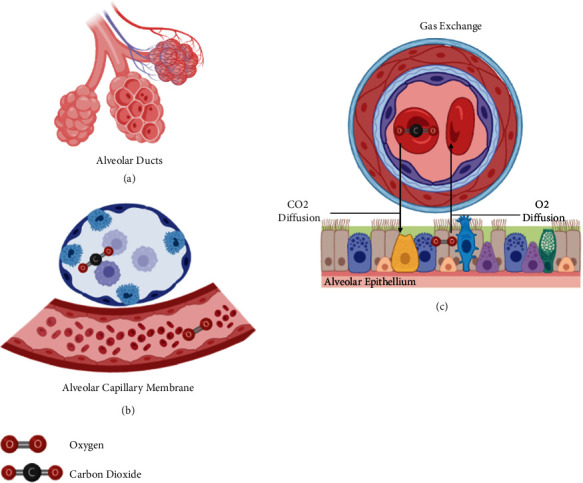
Pulmonary physiology: gas diffusion. When the gas reaches the alveolus (a), the oxygen diffuses through the alveolar-capillary membrane (b). Carbon dioxide diffuses from plasma to the alveoli and gets eliminated on exhalation (c). Figure adapted from [16].

During the diffusion process, in addition to the thickness of the alveolar-capillary membrane, it is crucial to see the differences in capillary pressures, determining that O_2_ uptake, for example, occurs in 2 stages. The first stage corresponds to the diffusion of O_2_ through the blood-gas barrier (plasma and erythrocytes); the second refers to the reaction of O_2_ with hemoglobin, in such a way that the definition of diffusion capacity is the gas flow divided by the pressure difference [17] (Figure 2).

The partial pressure of carbon dioxide levels represents the amount of CO_2_ dissolved in blood. The partial pressure of carbon dioxide is affected by VA, CO_2_ production, and a fraction of inspired CO_2_. An important lung function is to transmit air from the atmosphere to the gas exchange units of the lung, including the alveoli, alveolar sacs, alveolar ducts, and respiratory bronchioles. The rate at which air reaches the gas exchange units of the lung is known as the VA rate, which the respiratory rate (f) and VD^1^ influence (Table 1, Figure 2).

Alveolar ventilation means exhaling alveolar air to the environment, defined as the expired minute volume that reaches the alveoli, which is minute ventilation (VE) and the relationship between VD and tidal volume (VT) [18]. The human body is adapted and capable of eliminating CO_2_ as the body produces it in excess; unless there is a significant loss of pulmonary ventilation, metabolic processes will not induce hypercapnia [1, 18].

The compliance of the rib cage influences minute volume [13]. Thus, despite an adequate central respiratory drive and peripheral muscle function, limitations in lung expansion may occur [13]. These limitations lead to an increase in the compensatory respiratory rate (RR) that will result in fatigue that fails to increase total ventilation enough to compensate, leading to hypercapnia [13]. These relationships indicate that RR and VT are the two components of ventilation regulated by central and peripheral receptors, which are physiologically or artificially controlled to moderate CO_2_ elimination [13].

The total VD will be given by the sum of the anatomical and physiological dead space, referring to air that fills an airway but does not generally participate in gas exchange [13]. It comprises the nasopharynx, oropharynx, trachea, bronchi, bronchioles, and the alveolar space; when these do not receive adequate perfusion, the latter dramatically impacts systemic levels of CO_2_ and O_2_ [1, 13]. Intrinsic lung diseases that increase dead space are responsible for most cases of hypercapnic respiratory failure, while a smaller proportion is due to extrapulmonary conditions. The alveolar ventilation rate significantly impacts systemic CO_2_ and oxygen levels. Most commonly, PaCO_2_ levels get altered in response to changes in ventilation, respiratory depression (i.e., due to sedatives or narcotics), airway obstruction, and increased VD [1, 13].

The general impairment of alveolar ventilation cannot overcome CO_2_ production, resulting in hypercapnia, explained by the VA equation [19]. Based on this equation, an increase in respiratory rate should result in a reciprocal decrease in PaCO_2_. However, an increase in this parameter can shorten the overall inspiratory time, worsening dynamic hyperinflation and hypercapnia, as well as increased intrathoracic pressure that can result in hypotension, pneumothorax, and barotrauma [20–23].

## 3. Epidemiology: Frequency of Hypercapnia in Critical Patients

Hypercapnia is a frequent complication in critically ill patients and is associated with high morbidity and mortality. Ahmed et al. reported that 53% of patients treated with noninvasive mechanical ventilation for hypercapnic acute ventilatory failure died during hospitalization [24]. Another study looking at patients discharged from the ICU after an episode of acute hypercapnic ventilatory failure reported that readmission of 46% occurred within the first month, and 17% died within 12 months [25].

Hypercapnic ventilatory failure in patients with neuromuscular disorders represents 10% of all ICU admissions [26]. Patients with multiple comorbidities related to the development of hypercapnia, such as obesity and tobacco use [27], chronic obstructive pulmonary disease, or sleep-disordered breathing, may be hospitalized for pneumonia, hypertensive crisis, or decompensated heart failure, these being events that favor the appearance of hypercapnia and adverse outcomes [27, 28]. Multimorbidity contributes to the appearance of severe hypercapnic respiratory failure that requires ICU management and becomes one of the main predictors of mortality [29, 30].

## 4. Effects of Hypercapnia Oriented to the Critical Patient: the Good, the Bad, and the Ugly

The increase in CO_2_ produces a series of actions in the body with different physiological effects and clinical manifestations.

### 4.1. Cerebrovascular

The partial pressure of carbon dioxide levels and associated changes in pH impact arterial vascular tone and increase cerebral blood flow by 1-2 ml/100 g/min for every 1 mmHg increase in PaCO_2_ [31], which can cause cerebral edema and intracranial hypertension [32]. It is worth mentioning that hypocapnia also has deleterious effects described as ischemia and neuronal injury [33]. In addition, a recent study showed an association between hypocapnia and in-hospital death [34]. On the other hand, restoring normocapnia after sustained hypocapnia can result in normocapnic acidosis due to relative bicarbonate deficiency due to no compensation at the renal level [35].

Hypercapnia also generates increased sympathetic tone and decreased parasympathetic tone. Although there is still no clarity on the production mechanism, it may be due to increases in brain glutamine and gamma-aminobutyric acid, as well as reductions in glutamate and aspartate that act at the mid-level depressing VE and inspiratory work [36].

A multicenter, retrospective study in patients with brain injury (trauma, postarrest, and cerebrovascular event) between 2000 and 2015 reported that hypercapnic acidosis (HA) was associated with an increased risk of hospital mortality in 30,742 patients. However, when compensated to normal pH during the first 24 hours of ICU admission, hypercapnia might not be harmful in brain-injured patients undergoing mechanical ventilation [37].

There is a very narrow safety range of CO_2_ for brain protection; therefore, hypocapnia and hypercapnia can be harmful, increasing comorbidities and worsening the prognosis of patients.

### 4.2. Cardiovascular

Hypercapnic acidosis inhibits cardiac contractility and reduces systemic vascular resistance [38]. The net impact of mild hypercapnia is an increase in cardiac output through activation of sympathoadrenal mechanisms and sympathetic tone, increasing preload, decreasing afterload, and increasing contractility, heart rate, capacitance, and venous return [38]. Depending on the severity, it can progress to hemodynamic instability, fatal arrhythmias, and death. On the other hand, hypercapnia can produce cardiovascular depression with direct inhibition of cardiac and smooth muscle cell contractility, independently of pH levels [39].

### 4.3. Immune System and Inflammation

Hypercapnic acidosis suppresses innate and adaptive immune responses, specifically reducing neutrophil migration to septic foci while inhibiting phagocytosis. In addition, it impairs the release of proinflammatory cytokines by inhibiting hypercapnia; it selectively prevents the expression of interleukin-6 (IL-6) and tumor necrosis factor (TNF) [40]. On the other hand, it can influence lymphocytes and natural killer cells and affect phagocytosis by decreasing the macrophage cell line and alveolar macrophages, while the inhibition of phagocytosis occurs independently of hypoxia. Likewise, pH levels less than or equal to 7.20 are associated with increased bacterial proliferation in infectious models, increasing the growth of germs such as *Escherichia coli* and other bacterial species [41–43].

Other effects include the reduction of energy metabolism evidenced by the inhibition of the P65 protein pathway responsible for repair, proliferation, and growth at a cellular level, which makes it susceptible to apoptosis and suppression of free radical production [44].

### 4.4. Metabolic: Renal and Ionic

The kidneys are acutely sensitive organs to changes in PaCO_2_ [45]. Hypercapnia generates an increase in the secretion of hydrogen ions and the reabsorption of bicarbonate and sodium, which at a vascular level produces an alteration in blood flow directly by stimulating renal vasoconstriction and indirectly by systemic vasodilation secondary to high levels of PaCO_2_, which induces a drop in blood pressure and renal perfusion by activating the renin-angiotensin-aldosterone system [46, 47].

These changes observed with hypercapnia are independent of the changes seen in toxemia. The long-term effect of hypercapnia, caused by long-standing lung disease, is reduced glomerular filtration. Frank hypercalcemia by this mechanism is rare.

### 4.5. Respiratory

First, hypercapnia improves lung compliance through surfactant-independent mechanisms of actin-myosin interaction at the level of the lung parenchyma and increases pulmonary vascular resistance by enhancing hypoxic vasoconstriction [48, 49]. Regarding diaphragmatic function, certain studies show that patients with hypercapnia and spontaneous ventilation present dysfunction secondary to alterations in electrical signals of the afferent pathways of the phrenic nerve [50].

Hypercapnic acidosis affects cell membrane repair and alveolar fluid clearance [43] and suppresses the immune response [51]. Since most acute respiratory distress syndromes (ARDS) result from a lung infection, these effects [52] are vital [51], as they could worsen lung injury and spread distant organ failure.

## 5. Causes of Hypercapnia in the Ventilated and Nonventilated Critically Ill Patient

Once hypercapnia is suspected, it is necessary to carry out a comprehensive clinical assessment, ruling out risk factors and a complete physical examination to determine its possible cause [53]. For example, while looking for signs of imminent ventilatory failure that warrant the protection of the airway, it is essential first to take arterial blood gases and paraclinical tests that evaluate the internal environment and complete blood count [53].

**Figure 3:**
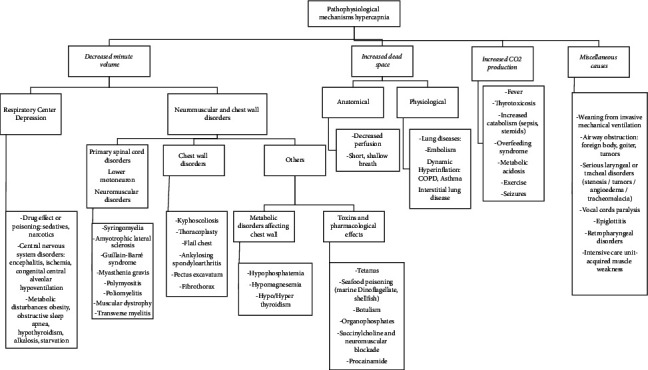
Pathophysiological mechanisms hypercapnia. Source: self-made.

There is no sensitive or specific imaging study for hypercapnia; however, taking a chest X-ray is helpful to rule out anatomical alterations, infectious pathologies, and pulmonary edema [53]. The metabolic, toxicological, cardiovascular, central nervous system, and neuromuscular disorders, among other extension studies, are requested according to the suspected diagnosis (Figure 3).

Hypermetabolic states such as sepsis, malignant hyperthermia, thyrotoxicosis, and overfeeding can elevate CO_2_ levels. The presence of fever significantly impacts PaCO_2_ levels, with each degree Celsius increase in core body temperature increasing CO_2_ production by 14% [8, 54].

The administration of sodium bicarbonate in a clinical setting can result in the generation of CO_2_. Patients requiring dialysis are commonly administered citrate-containing anticoagulants, as citrate metabolizes into bicarbonate in the liver, which combines with hydrogen ions produced by organic acids and dissociates to produce CO_2_. Citrate may contribute to an increase in PaCO_2_ [54].

While in nonventilated patients, the fraction of inspired CO_2_ is negligible, and critically ill patients who are on mechanical ventilation can become hypercapnic through rebreathing of CO_2_ that accumulates in the respiratory circuit [55]. Although healthy individuals regulate blood CO_2_ levels through negative feedback mechanisms modulated by central and peripheral chemoreceptors [2, 3], hypercapnia can be intentionally or inadvertently induced in patients requiring mechanical ventilation [54, 55].

In patients with acute respiratory distress syndrome, where the work of breathing increases CO_2_ production by up to 30%, there is an increased demand for O_2_ on the respiratory muscles, producing tachypnea and imminent ventilatory failure, requiring invasive mechanical ventilation as a means of support [56].

Noninvasive ventilation (NIV) is usually indicated in patients with hypercapnia with respiratory acidosis (pH < or equal to 7.35), as has been elegantly summarized by the most recent guidelines [57]. Regarding the use of NIV in neuromuscular diseases, its initiation is recommended in the presence of symptoms of hypoventilation accompanied by PaCO_2_ levels >45 mmHg, oxygen saturations <88% for at least 5 minutes, forced vital capacity <50% of the theoretical value in a supine or upright position, and maximal inspiratory pressure ≥60 cm H_2_O, as indicators of diaphragmatic weakness [58]. A recently published survey showed that although these are the major indications, there are other important parameters that need to be considered [59]. In these patients, bilevel NIV with fixed or self-adjusting pressure with low expiratory positive pressure (EPAP) can be used to improve CO_2_ elimination [58].

Mechanical ventilation with parameters of lung protection or less harmful has shown a significant benefit in survival [60]. Such a strategy has been associated with a progressive reduction in VT in recent decades [61, 62], bringing a progressive increase in PaCO_2_ levels. One of the great clinical questions, fundamental to resolving in our patients, is to define to what extent low VT are protective when they generate hypercapnia, which, in turn, is associated with increased morbidity and mortality [54, 63].

Permissive hypercapnia in patients receiving lung-protective mechanical ventilation can occur in two contexts: (1) hypercapnia and acidosis, where there are elevated CO_2_ levels with lower pH, and (2) hypercapnia with normal pH. The latter may be present in states of volume contraction or metabolic compensation for respiratory acidosis [64]. It is unclear whether HA leads to survival benefits as an independent factor of lung-protective ventilation.

Hypercapnic acidosis can reduce protein leakage, pulmonary edema, and inflammation and protect against free radical-mediated injury while preserving lung compliance [7, 64]. When we counteract this effect, it leads to higher levels of permeability and oxidative damage due to increased xanthine oxidase activity [65, 66].

In two randomized controlled trials evaluating lung-protective ventilation, PaCO_2_ levels remained higher in patients receiving low-VT ventilation, even though they had an equivalent minute volume to the conventional group [60, 67]. There were no differences in pH for both groups after 36 hours, possibly due to buffering agents or metabolic compensation. A secondary analysis of the ARDS network showed a reduction in mortality with VT of 6 ml/kg, not with VT of 12 ml/kg. The investigators' theory was that ventilator-induced lung injury occurred to a lesser extent in subjects receiving VT 6 ml/kg. Some patients may generate lung injury even at low VT; thus, a VT personalization strategy is suggested to decrease this risk [60, 67].

## 6. Permissive Hypercapnia: Evidence

One of the essential concepts in critical care has been the recognition of mechanical ventilation as supportive therapy in patients with respiratory failure. However, such therapy can worsen or even cause lung injury, multiple organ failure, and death in what is known as ventilator-induced lung injury (VILI). Protective ventilation with low VT and the consequent elevation of the partial pressure of carbon dioxide has been called “permissive hypercapnia” [7]. Over the years, clinical studies have evaluated a progressive increase in PaCO_2_ levels (Table 2).

To our knowledge, no extensive clinical studies evaluate the effect of acute hypercapnia or compensated hypercapnia in patients undergoing mechanical ventilation. Two large series can guide the impact of hypercapnia on mortality.

A secondary analysis of three cohort studies in 40 countries, performed on 1,899 patients with ARDS, reported that a PaCO_2_ ≥ 50 mmHg had more complications, organ failure, and worse outcomes. Thus, hypercapnia was associated with higher mortality in the ICU (OR 1.93, CI 1.32–2.81;*p*=0.001) [56]. A multicenter, binational, retrospective study that included 252,812 patients showed that HA and compensated hypercapnia were associated with increased in-hospital mortality, extended ICU stay, and prolonged hospitalization [68].

## 7. How to Manage Hypercapnia

In hypercapnic respiratory failure associated with hypoxemia, the administration of supplemental oxygen is essential. According to the alterations at the level of the sensorium and the arterial blood gas, the device will be defined, among which are assistance with noninvasive positive pressures such as CPAP and BiPAP or orotracheal intubation with invasive ventilation [53]. The main objective of oxygen therapy in these patients is the management of hypoxemia, while the secondary objective is to avoid a clinically significant worsening of hypercapnia [53].

The prone position unloads the right ventricle and redistributes the VILI, improves oxygenation and compliance, and, when performed early, improves prognosis and mortality [69–71]. For effects of hypercapnia when a patient is in a prone position, it is necessary to monitor PaCO_2_ reduction instead of PaO_2_, which may better reflect the degree of pulmonary recruitment [72, 73].

Protective mechanical ventilation can result in the production of hypercapnia, but it can also be the result of increased dead space. Early hypercapnia in the context of ARDS results in increased mortality [74]. To reduce this risk, some authors have proposed various strategies or interventions [56, 75–77]:

1. Look for optimal PEEP levels (PEEP-FiO_2_ titration), avoiding alveolar overdistention
2. Look for optimal tidal volume 6–8 ml/kg, limit 10 ml/kg
3. PaCO_2_ threshold 50 mmHg, upper limit 70 mmHg
4. Target PaO_2_ 55–80 mmHg to avoid high FiO_2_
5. Find optimum driving pressure
6. In increases in RR to correct hypercapnia, monitor tolerance to reduce the risk of dynamic hyperinflation and significant right ventricular dysfunction
7. Low plateau pressure was associated with driving pressure when PaO_2_/FiO_2_ < 150 had shorter mechanical ventilation times
8. Prone position
9. Low tidal volumes
10. Monitor lung compliance
11. Permissive underfeeding 40–60%
12. Mechanical power
13. BICARB-ICU protocol if PH < 7.20, PaCO_2_ < 45 mmHg, HCO_3_ < 20 mmol/L, and lactate > 2 mmol/l start sodium bicarbonate solution at 4.2% volumes of 125–250 ml in 30 minutes maximum 1 l per day to maintain PH > 7.30

Using a buffer remains a controversial issue for patients with hypercapnic acidosis. HA is only beneficial when associated with low VT [78]. Deep metabolic and hypercapnic acidosis with pH levels <7.10 are associated with adverse physiological effects [44, 79], to the point that severe acidosis can impair myocardial contractility and reduce cardiac output, leading to refractory hypotension. Other effects caused by severe acidosis are the alteration of the mental sphere, immune function, and reduction of energy metabolism [79].

Studies with bicarbonate infusions have shown no benefit in improving pH and suggest many treatment-related adverse effects, including hypervolemia, hyperosmolarity, and increased lactate levels [79]. The ARDS network clinical trial and the ARMA study allowed bicarbonate infusions when the pH fell below 7.15 [60, 80–82].

In the available evidence concerning the mortality vs. CO_2_ level outcome, it is noteworthy that in studies using bicarbonate to control acidosis, there was less variation in PaCO_2_ levels, but there is no clear benefit in mortality [60, 75–77, 81]. The efficacy of bicarbonate infusion depends on the ability to excrete carbon dioxide; this ability is physiologically maintained at a pH > 7.20 in such a way that there could be a benefit with bicarbonate solutions when the pH is above this level, an effect that was demonstrated in the BICARB-ICU protocol having CO_2_ as limiting [43]. Finally, bicarbonate infusion can worsen cellular acidosis by passively diffusing into cells and reacting with carbonic anhydrase to produce carbonic acid [44, 79].

The use of tris-hydroxymethyl aminomethane may be helpful to buffer HA by buffering cell pH and reducing CO_2_ levels. It may also mitigate the adverse effects of acidosis on the cardiovascular system and restore hemodynamic stability [83]. Further studies are required to standardize its use since it does not solve the problem of nonperfused regions of the lung.

Finally, refractory respiratory acidosis is considered a criterion for starting extracorporeal life support (ECCO_2_R) [60, 84]. ECCO_2_R is a novel technique to eliminate CO_2_ through a venovenous bridge without affecting oxygenation [85]. To date, it is used in refractory acidosis or as a treatment for complicated respiratory acidosis in low VT lung-protective ventilation with permissive hypercapnia; further studies are needed to standardize its routine application in the treatment of hypercapnia [85].

## 8. Conclusion

Although most respiratory insufficiencies are hypoxemic, acute hypercapnic respiratory failure is becoming more frequent. Pneumonia commonly causes hypoxemic ventilatory failures, and hypercapnic ventilatory failures are primarily due to intrinsic pulmonary diseases, which increase dead space, such as COPD, while a smaller proportion is due to additional lung conditions. Protective ventilation with low volumes and, amid the pandemic, the management of advanced phases of ARDS increased the frequency of patients with hypercapnia. It is essential to know the triggering factors, the pathophysiology, and the impact on clinical outcomes to provide therapy directed at the primary cause and avoid possible complications.

## Figures and Tables

**Table 1:** Respiratory physiology formulas.

Name	Formula
Diffusion of simple gases, Fick	*Gv*=*A∗D∗*(*P*1 − *P*2)/*T*
Alveolar ventilation	*VA*=*VE* − *V* *D*
	VA=VCO_2_*∗*K/PaCO_2_
Minute volume	*VE*=*FR* × *VT*
Tidal volume	*VT*=*VE*/*FR*
Alveolar O_2_ pressure	PAO_2_=FiO_2_*∗*(Patm − PH_2_O) − (PCO_2_/R)
Cellular respiration	C_6_H_12_O_6_+6O_2_=6CO_2_+6H_2_O
CO_2_ blood pressure	PaCO_2_=*K∗*(VCO_2_/*VA*)
Acid-base balance	PH=6,1+log(HCO_3_−/0,03*∗*PCO_2_)
	CO_2_+H_2_0=H_2_CO_3_=H+HCO_3_

GV, gas volume; A, exchange surface; *D*, diffusion constant; P1−P2, partial pressure difference (of 2 gases or one same gas in two different areas); T, thickness; VA, alveolar ventilation; VE, minute volume; VD, dead space; RR, respiratory rate; VT, tidal volume; PaO_2_, alveolar pressure of oxygen; FiO_2_, fraction of inspired oxygen; Patm, atmospheric pressure; PH_2_O, water vapor pressure; PaCO_2_, pressure arterial CO_2_; R, respiratory quotient (0.8); C_6_H_12_O_6_, glucose; K, constant (863); VCO_2_, volume of CO_2_; HCO_3_, bicarbonate. Adapted from [17].

**Table 2:** Evolution of PaCO_2_ levels in a cohort of critically ill patients under mechanical ventilation.

Year	Study	Patients	PaCO_2_ level (mmHg)
2016	Lung safe	29.1400	46 (45.1–46.6)
2015	eICU	4,361	41 (35–48)
2013	PROSEVA	237	Control: 47 ± 14; intervention: 45 ± 9
2012	MIMIC-III	3,846	39 (34–44)
2010	ACURASYS	340	Control: 44 ± 9; intervention: 45 ± 11
2000	ARDS network	861	Control: 35 ± 8; intervention: 40 ± 10
